# Mapping seasonal dynamics of forage and cereal crops in a hyper-arid environment using Sentinel-1 and Sentinel-2 time series

**DOI:** 10.3389/frai.2026.1803320

**Published:** 2026-07-24

**Authors:** Areej Alwahas, Kasper Johansen, Jorge Rodriguez, Matthew F. McCabe

**Affiliations:** Division of Biological and Environmental Science and Engineering (BESE), King Abdullah University of Science and Technology, Thuwal, Saudi Arabia

**Keywords:** crop-type mapping, irrigated agriculture, multimodal time series, pseudo labels, self-supervised learning

## Abstract

**Introduction:**

In arid and hyper-arid regions, agriculture depends heavily on irrigation, making crop type monitoring important for water allocation, monitoring crop management policies, and providing the information required to forecast food supply. However, field labels are often scarce, and crop calendars can shift due to locally managed planting, harvest, and irrigation decisions, complicating mapping at field-scale.

**Methods:**

We present a seasonal crop type mapping approach applied to Wadi Al-Dawasir, Saudi Arabia, generating maps for 2020–2024 from biweekly optical and radar satellite time series. The method learns representations from unlabeled imagery through self-supervised pretraining and fine-tunes a segmentation model using a small set of field observations with pseudo-label augmentation from unsupervised clustering. We mapped four classes: fallow, cereal, vegetables, and forage, and evaluated performance using overall accuracy, F1-score, and mean intersection-over-union (mIoU).

**Results:**

The configuration combining self-supervised pretraining, pseudo-label augmentation, and optical-radar inputs achieved an mIoU of 0.80, an F1-score of 0.88, and an overall accuracy of 0.97. In contrast, using optical information alone substantially reduced accuracy, with an mIoU of 0.35, an F1-score of 0.32, and an overall accuracy of 0.64. Fallow and forage produced the highest mapping accuracies, with mIoU values of 0.97 and 0.85, respectively, while vegetables had the lowest accuracy among the four crop groups, with an mIoU of 0.52.

**Discussion:**

These results show that self-supervised temporal pretraining combined with pseudo-label augmentation can support efficient multi-season, field-scale crop mapping using limited labels in irrigation-driven arid regions. The lower accuracy for vegetables highlights the continued challenge of mapping heterogeneous crop groups with overlapping phenological patterns.

## 1 Introduction

Agricultural landscapes are changing in response to climate pressures, water scarcity, and policy interventions, creating a need for robust crop monitoring systems (Food and Agriculture Organization of the United Nations, 2021; Food and Agriculture Organization of the United Nations and Global Information and Early Warning System, 2024; IPCC, 2022; OECD, 2023; Potapov et al., 2022). Cereal crops alone account for approximately 56% of global dietary energy and about 50% of global dietary protein (Muitire et al., 2021), with wheat, rice, and maize making up the majority of global calorie intake (Food and Agriculture Organization of the United Nations, 2023; Food and Agriculture Organization of the United Nations and Global Information and Early Warning System, 2024; World Bank, 2025). On the other hand, forage crops, including alfalfa and other grasses, are used primarily as livestock feed, supporting the production of dairy and meat (Joseph et al., 2025; Vendramini et al., 2023). The production of these staple cereals is concentrated in a relatively small group of countries, with maize being predominantly produced in the United States, China, and Brazil, rice in China, India, Indonesia, and Bangladesh, and wheat in China, India, Russia, and the United States (Food and Agriculture Organization of the United Nations, 2023; World Bank, 2025). While forage crops such as irrigated alfalfa are widely cultivated across diverse agricultural systems.

Observing the pattern and development of crop systems is critical for the planning and management of global food systems (Becker-Reshef et al., 2020). For instance, the seasonal dynamics of cereal and forage crops, including planting and harvest timing, number of forage cuts, and peak greenness, can be observed from satellite image time series, revealing crop responses to environmental conditions, crop management practices, and water availability (Li G. et al., 2023; Meroni et al., 2021; Veloso et al., 2017; Zhang et al., 2019). More broadly, monitoring seasonal dynamics is essential to predict market trends, manage food security goals, and improve irrigation strategies (Becker-Reshef et al., 2019; Calera et al., 2017).

In response to the increasing global demand for food and livestock feed, and the desire to improve food self sufficiency, production of cereal and forage crops has expanded into arid and hyper-arid regions of the Middle East, North Africa, and Central Asia (Hamidov et al., 2016; Li T. et al., 2023; Potapov et al., 2022). However, this intensification has increased pressure on scarce groundwater resources used for irrigation, many of which are non-renewable and associated with unsustainable depletion in drylands (Siebert et al., 2010; Voss et al., 2013; Wada et al., 2010). Saudi Arabia is an extreme example of this trend, with considerable cereal and forage farming taking place in one of the world's most arid and water-stressed regions (Abdelbaki and Alzahrani, 2024; Li T. et al., 2023). Such a context creates a clear need for robust, spatially explicit monitoring systems to evaluate how cropping patterns are changing under water stress and policy interventions (Becker-Reshef et al., 2020; Van Tricht et al., 2023).

Satellite-based remote sensing combined with machine learning and deep learning offers cost-effective solutions for crop monitoring that are scalable and repeatable (Burke et al., 2021; Nakalembe et al., 2021). Sentinel-1 and Sentinel-2 image time series have been used to capture agricultural phenological stages, identify crop types, and delineate field boundaries (Masoud et al., 2020; Tian et al., 2021; Veloso et al., 2017). However, many supervised approaches depend on sufficient and accurate reference data, which are difficult and expensive to collect repeatedly over large areas, particularly in data-scarce regions (Taylor et al., 2021). Existing global efforts, including WorldCereal and Global Food Security Analysis Data (GFSAD), provide seasonal crop information at 10 m and 30 m resolution, respectively (Van Tricht et al., 2023; Yadav and Congalton, 2018). Yet arid regions remain underrepresented in global datasets, and crop mapping models often underperform in irrigation-driven environments that differ from temperate rainfed systems (Taylor et al., 2021; Thenkabail et al., 2021; Van Tricht et al., 2023).

In arid and hyper-arid systems, relatively few studies have demonstrated reliable discrimination of major crop types using satellite imagery. Recent studies in the Nile Delta and central Iran have shown that multi-temporal Sentinel-1 and Sentinel-2 data can support mapping of wheat, barley, irrigated forage, and other major crops, but they also report persistent constraints related to sparse and uneven ground truth data, irregular phenology, and weak transferability across seasons or neighboring regions (Asgarian et al., 2016; Mobarakeh et al., 2025; Saleh et al., 2025; Taylor et al., 2021; Van Tricht et al., 2023; Wang et al., 2019). These constraints motivate label-efficient approaches that reduce reliance on dense ground truth data, including self- or semi-supervised representation learning and the use of pseudo labels (Ma et al., 2022; Xu et al., 2024). In irrigated drylands, pseudo labels can also be informed by recognizable field geometry, such as center pivot patterns, while time series inputs allow evaluation without relying on fixed calendar rules (Johansen et al., 2021; Li T. et al., 2023).

Over recent decades, Saudi Arabia's irrigated agriculture has shifted in response to policy, water scarcity, and climate variations (Li T. et al., 2023; Odnoletkova and Patzek, 2023). Since 2018, reforms under the National Water Strategy, such as restrictions on water-intensive crops, water pricing, and subsidy changes, have created clear shifts in agricultural activity and cropping trends on the ground. These efforts also exposed a significant gap in consistent agricultural monitoring (Food and Agriculture Organization of the United Nations and Global Information and Early Warning System (FAO GIEWS), 2024; McIlwaine and Ouda, 2020; Ministry of Environment, Water and Agriculture, 2018; US-Saudi Business Council, 2022). In particular, there is a lack of seasonal, field-scale crop type maps that can track how cropping patterns and intensity respond to policy initiatives for irrigated crops. Here, we address this gap by developing and evaluating a satellite-based time series approach in an agricultural region with limited ground truth data availability (Alqarawy et al., 2019; Masoud et al., 2020).

In this study, we developed and evaluated an approach for seasonal crop type mapping in Wadi Al-Dawasir, Saudi Arabia, using Sentinel-1 and Sentinel-2 image time series. The study was conducted in a label-scarce setting where crop phenology is irrigation-driven and irregular. We assessed whether self-supervised learning can support field-scale classification across multiple growing seasons with limited supervision. The analysis focused on four functional crop groups, fallow, cereal, vegetables, and forage, from 2020 to 2024. The main contribution is a field-level crop type mapping workflow designed for a label-scarce, irrigation-dependent agricultural region in Saudi Arabia. It combines self-supervised temporal learning, pseudo label augmentation, and optical–radar time series to reduce reliance on dense field labels while preserving crop phenology and field structure.

## 2 Data and methods

### 2.1 Study area

**Figure 1:**
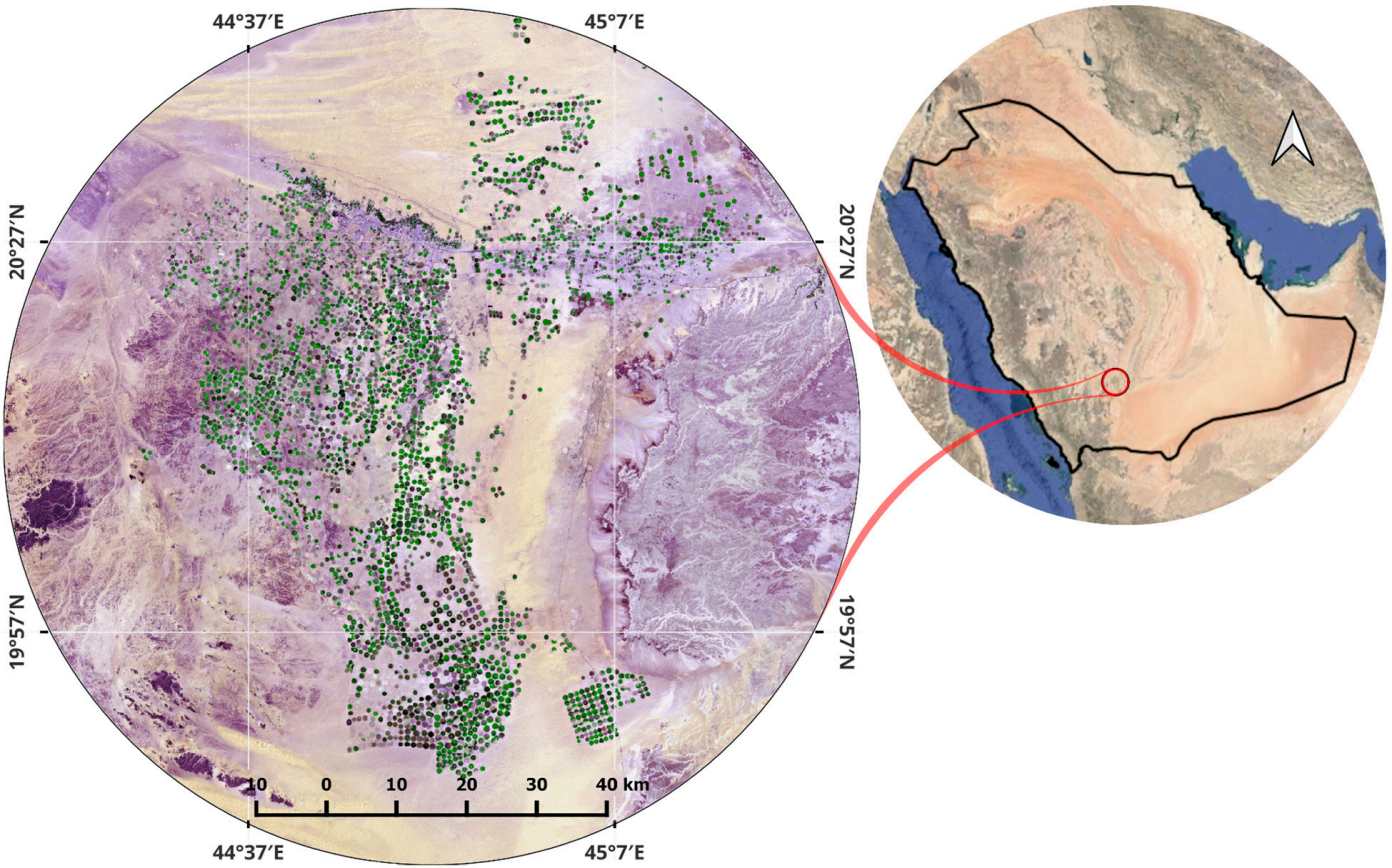
Study area of Wadi Al-Dawasir, Saudi Arabia.

The study area focuses on Wadi Al-Dawasir in Saudi Arabia, which covers about 4,560 km^2^ (76 km × 60 km) in and is near the southernmost part of the Najd Plateau between latitudes 20° and 21° N and longitudes 44° to 46° E, shown in Figure 1. The climate is hyper-arid, with the area receiving an annual average of less than 50 mm of rain that tends to be concentrated in the winter months (Abdelbaki and Alzahrani, 2024; Alqarawy et al., 2019). Reported diurnal temperatures of typical daily minimum and maximum conditions in winter temperatures drop to around 6 °C, and summer temperatures occasionally reach 45 °C (Masoud et al., 2020), resulting in extremely high evaporation rates and frequent windstorms, all of which contribute to soil degradation. Wadi Al-Dawasir is known for its sand dunes and flat terrain punctuated by valleys. Deep non-renewable subsurface aquifers are the primary source for irrigation in this region (Ministry of Environment, Water and Agriculture, 2023; Morton, 2019).

Agriculture in Wadi Al-Dawasir is almost entirely irrigated and relies on large scale farming supported by center pivot irrigation systems (Abdelbaki and Alzahrani, 2024; Li T. et al., 2023). Cereal crops such as wheat and barley, as well as forage crops like lucerne and Rhodes grass, are crucial for cattle feed and are produced at scale. A range of vegetables, such as potatoes, onions, and chili peppers, are also grown, particularly during the colder months (Alqarawy et al., 2019; Masoud et al., 2020). Although agriculture has placed significant strain on the extremely limited water resources and highlighted the vital need for more sustainable practices, food security related policies and government-supported infrastructure have mostly spurred agricultural expansion in the region (Abdelbaki and Alzahrani, 2024; Masoud et al., 2020).

### 2.2 Data

#### 2.2.1 Satellite data

Optical and radar data from 2020 to 2024 were used to analyze irrigated fields in Wadi Al-Dawasir. Optical data included Sentinel-2 Level-2A surface reflectance images (*COPERNICUS/S2_SR_HARMONIZED*), which provide 13 spectral bands across the visible, red edge, near infrared (NIR), and shortwave infrared regions. The Normalized Difference Vegetation Index (NDVI) was derived from the red and NIR bands to characterize crop phenology. Sentinel 2 has a 5-day revisit period from the combined S2A and S2B satellites and a spatial resolution of 10–20 m (Drusch et al., 2012; European Space Agency, 2015). In this study, NDVI was used as a compact optical representation of crop phenology to reduce input dimensionality and computational cost under limited label availability. Using all Sentinel-2 bands across biweekly time steps would substantially increase the input size and memory requirements of the temporal model. However, we did not directly compare NDVI-only inputs with full multispectral Sentinel-2 inputs; therefore, this choice should be interpreted as a practical phenology-based design decision rather than an optimized spectral configuration.

Sentinel-1 radar imagery was acquired as Interferometric Wide Swath (IW) Ground Range Detected (GRD) scenes, with dual polarizations, i.e., VV and VH (*COPERNICUS/S1_GRD*). VH polarization is particularly sensitive to vegetation structure because of volume scattering effects, which makes it valuable for agricultural monitoring (Bauer-Marschallinger et al., 2021; Vreugdenhil et al., 2018). In this study, we focused on the use of the VH/VV ratio, as preliminary tests indicated that VV alone did not improve the classification accuracy. Sentinel-1A and 1B combined had previously ensured a 6-day revisit cycle, but since the decommissioning of Sentinel-1B in August 2022, only Sentinel-1A has been operational (until the launch of Sentinel-1C in December 2024), extending the revisit time to 12 days (European Space Agency, 2022). As our analysis was based on biweekly composites, this reduction in acquisition frequency did not affect the temporal resolution used. All imagery was accessed and processed in the Google Earth Engine (GEE), including cloud/shadow masking, temporal compositing, and Sentinel-1 and Sentinel-2 data alignment, are described in Section 2.3.1.

#### 2.2.2 Field data collection and preparation

**Figure 2:**
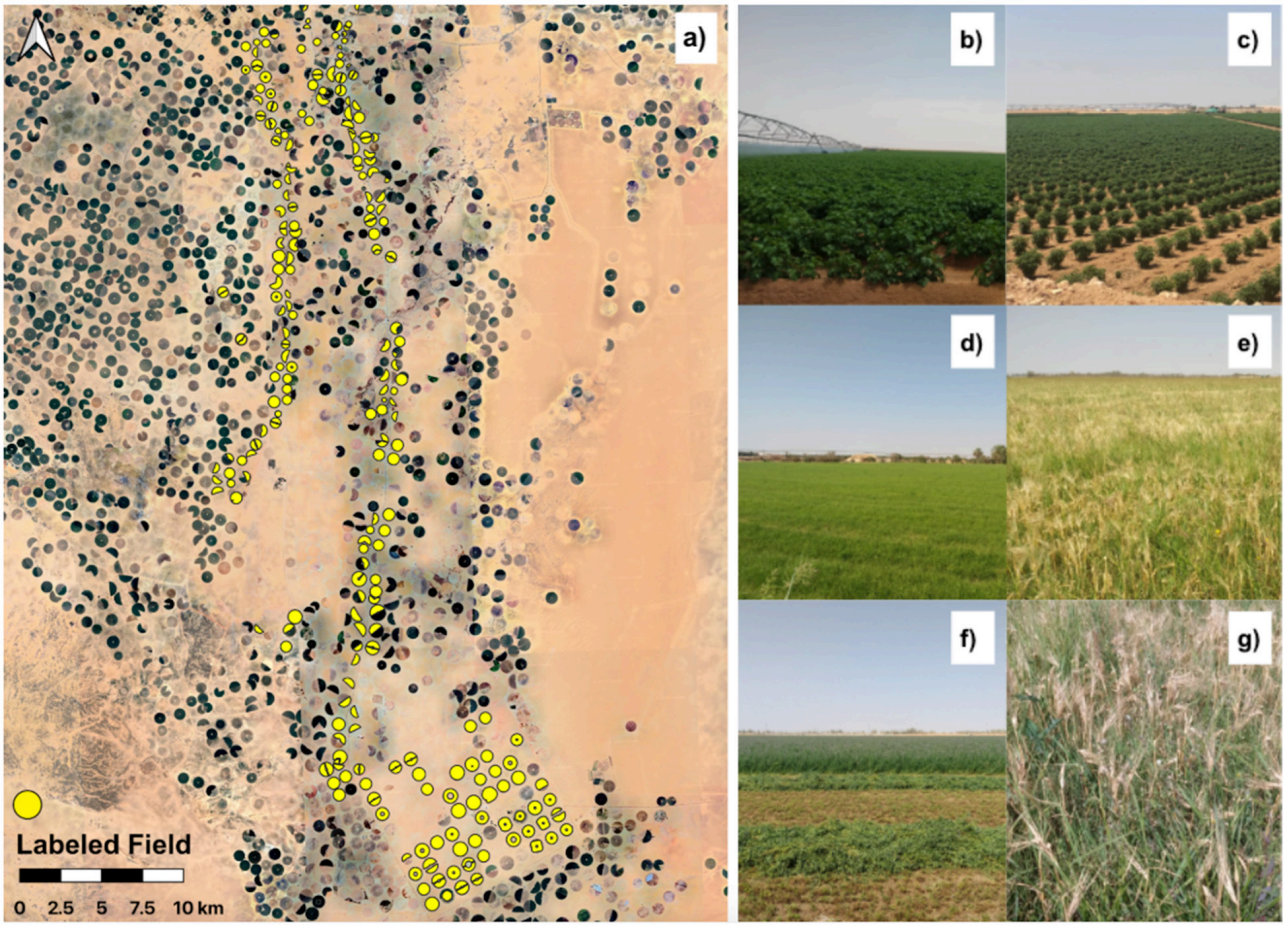
Visited fields and representative photographs from Wadi Al-Dawasir: **(a)** map of center pivot fields visited during the 2023–2024 campaigns (yellow circles); representative *in-situ* photographs of **(b)** potato, **(c)** chili pepper, **(d)** and **(e)** wheat, **(f)** alfalfa, and **(g)** barley.

Four field data collection campaigns were conducted in Wadi Al-Dawasir on March 16 to 21, 2023 (winter), September 10 to 15, 2023 (summer), January 31 to February 3, 2024 (winter), and September 5 to 10, 2024 (summer) to support the development and validation of satellite-based crop type models. The data collection covered the same 300 center pivot fields each visit. A Trimble handheld GPS-enabled device was used *in situ* for crop type labeling with the obtained data serving as the ground truth for evaluating and fine-tuning the developed models for crop type mapping. More fields were cultivated during winter, with some fields being fallow during the summer months. The same fields were visited to record the crop types and track field dynamics over the different growing seasons. Fields with no visible standing crop on the survey date were recorded as candidate fallow observations during field data collection. Final fallow labels for each field were assigned only after checking the corresponding Sentinel-2 NDVI temporal profile for the associated growing season to confirm persistently low vegetation activity (Section 2.2.4). Fields showing evidence of recent harvest, regrowth, or a short idle interval within an otherwise active seasonal cycle were excluded from the labeled dataset. To understand crop growth behavior in the satellite image time series, we recorded the phenological stage by visual observation following FAO definitions (vegetative, heading, flowering, senescence) and used these notes to compare the recorded phenological stage against the Sentinel-2 derived time series. Sentinel-2 images were used to manually delineate the boundaries of the 300 fields visited during the field campaigns using Quantum Geographic Information System (QGIS) (Figure 2).

#### 2.2.3 Field sample distribution

**Figure 3:**
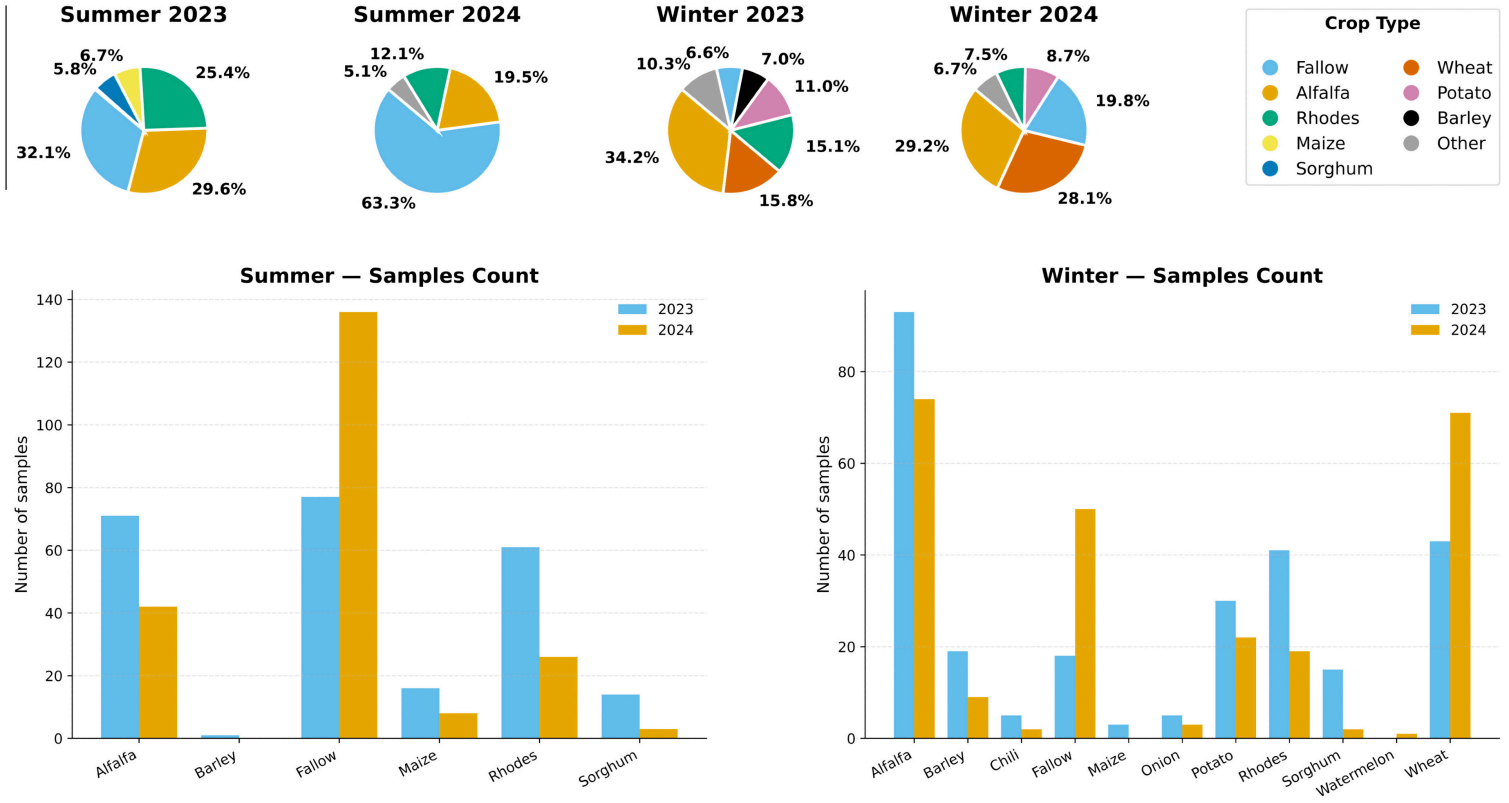
Distribution of crop types recorded during the 2023–2024 summer and winter field campaigns. Percentage share of sampled fields **(top)** and field counts by crop type **(bottom)**. “Fallow” indicates no crop on the survey day. Values reflect surveyed fields, not regional totals.

The collected field data of crop type varieties in Wadi Al-Dawasir across the 2023 and 2024 growing seasons and the seasonal trends are summarized in Figure 3. In winter, crops grown for human consumption included wheat, barley, onion, potato, and chili pepper. Forage crop production for livestock feed was dominated by alfalfa, with some fields also labeled as Rhodes grass and maize. The summer season was dominated by forage crops, including alfalfa, Rhodes grass, maize, sorghum, and a few barley fields. This seasonal pattern was affirmed in reports received from the farmers, who emphasized that summer crops mainly focus on livestock feed due to the harsh summer conditions. Rhodes grass was among the dominant crops, accounting for 25.4% during the summer of 2023. However, in 2024 its distribution declined to 12.1%, and fallow fields doubled from 32.1% in 2023 to 63.3% in 2024. A similar decline from 29.6% to 19.5% was observed for alfalfa fields between winter and summer.

Alfalfa remained a key crop during all seasons, but the number of fields decreased between the winter of 2023 from 34.2% to 29.2% in the winter of 2024. Wheat, on the other hand, increased from 15.8% in 2023 to 28.1% in 2024. Potatoes and other crops were present in both winters, with smaller proportional shares of around 10% or less. Since we revisited the same fields during each campaign, the varying numbers of fields per crop type show that farmers vary the crop types grown in the same fields.

#### 2.2.4 Label quality control

**Figure 4:**
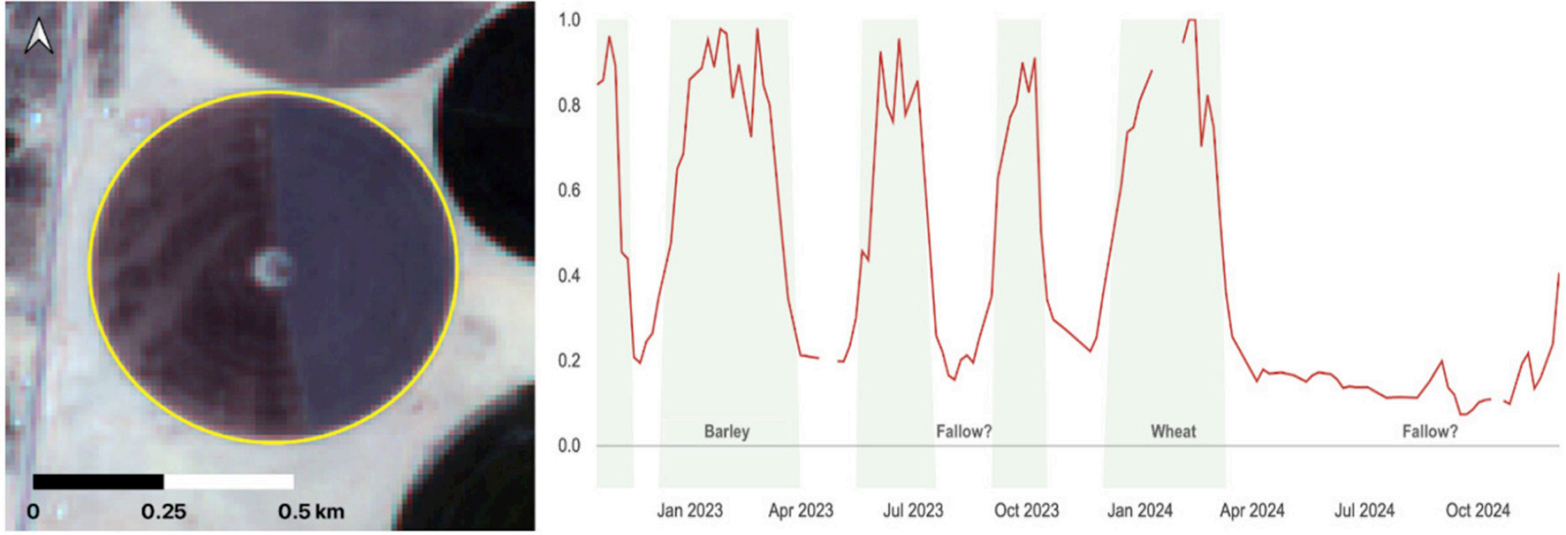
Representative center pivot field in Wadi Al-Dawasir **(left)** and its Sentinel-2 NDVI time series **(right)**, with the red line showing the NDVI curve.

To ensure label quality, all fields marked as fallow during the campaign were cross-checked against the Sentinel-2 NDVI time series around the time of visit, as illustrated in Figure 4. If the NDVI profile indicated active growth or a recent harvest, meaning that the field was only idle on the visit day, then the record was removed from the training/validation set. Figure 4 illustrates an example center pivot and its Sentinel-2 NDVI trajectory. The curve in Figure 4 exhibits a single pronounced peak between January and April 2023, consistent with a winter cereal (barley was observed during the winter field visit). In the summer season, between May and October 2023, the curve for the same center pivot shows repeated cut and regrow pulses, which are characteristic of irrigated forage. However, the field was visited in September, and it was recorded as fallow on that survey day. Therefore, to avoid misclassification errors due to a day specific observation that contradicts the surrounding NDVI dynamics, this data point was removed.

For the same center pivot, the NDVI rises again and reaches a single peak between approximately January and April 2024, consistent with a cereal cycle. In this case, wheat was observed during the corresponding field visit. This is followed by a prolonged low NDVI period from approximately May to October 2024, indicating fallow conditions during mid-to-late 2024. Because these field observations are consistent with the NDVI dynamics around the visit dates, the corresponding data points were retained. The same NDVI screening check was performed for every labeled center pivot field in the dataset, to remove observations that conflicted with the expected NDVI seasonal signal.

### 2.3 Methods

**Figure 5:**
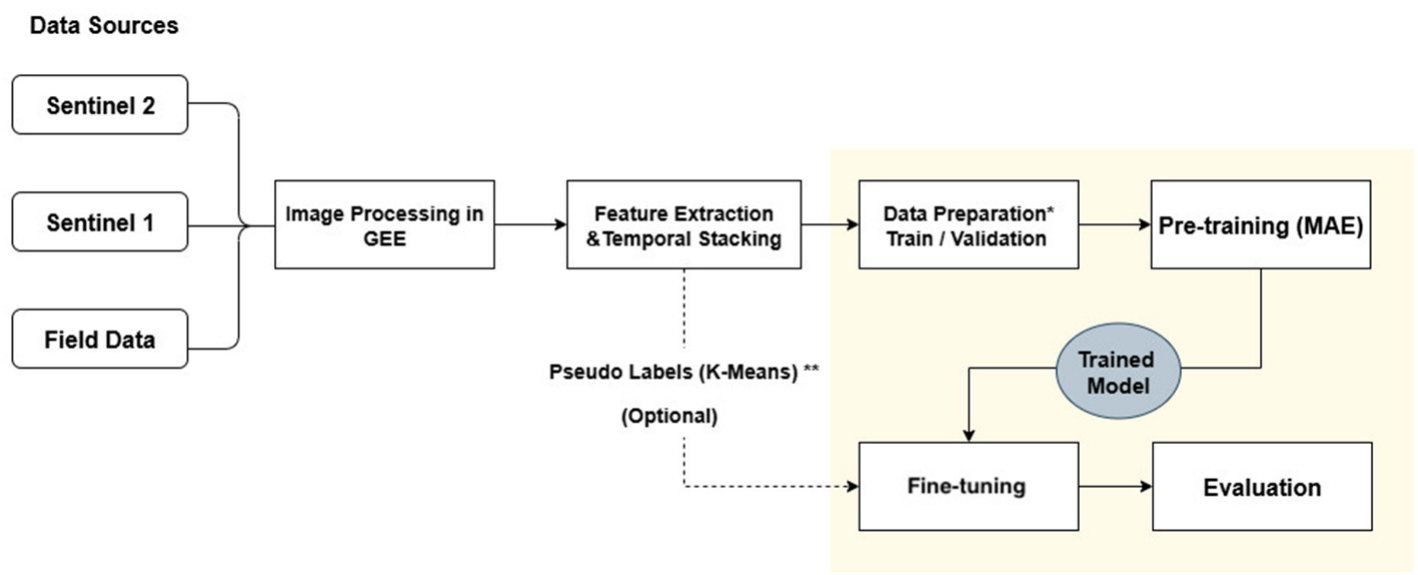
End-to-end pipeline showing the main steps used to produce seasonal, field-scale crop type maps.

Our approach for mapping crop types integrates multi-sensor inputs, i.e., NDVI from Sentinel-2 and VH/VV from Sentinel-1. The workflow consists of six stages: (i) image processing in GEE, (ii) creating biweekly time-series stacks of NDVI and VH/VV, (iii) preparing the data for modeling by post-processing, tiling, and splitting the data into train and validation subsets, (iv) pretraining the MAE model on unlabeled image time series, (v) fine-tuning with different supervision settings using verified field labels with optional K-means pseudo-label augmentation, and (vi) evaluation on a held-out subset using OA, UA/PA, F1, and IoU (Figure 5). Pseudo labels were generated separately using K-means clustering on the seasonal NDVI and VH/VV time-series inputs and were used only as auxiliary supervision during fine-tuning. They were not used for pretraining, validation, or model selection. The dashed branch in Figure 5 indicates this optional pseudo-label pathway.

The dataset was split into training and validation sets using a random, stratified split by crop class to ensure that all classes were represented in both subsets despite class imbalance. The validation subset was kept aside and was never seen by the model during the pre-training stage. We generated pseudo labels using a K-means approach, as detailed in Section 2.3.2, to augment our limited collected labels. In the pre-training stage, a masked autoencoder (MAE) was trained on the biweekly Sentinel-1/2 stacks to learn temporal representations (Section 2.3.3). For the supervised fine-tuning stage, the pre-trained encoders were updated using the crop type labels using two approaches: (1) a RF classification, and (2) a segmentation (DeepLabv3+) to produce the final crop type map (Section 2.3.4).

Mapping accuracy of crop types was assessed based on independent withheld field data and using the overall accuracy (OA), user's and producer's accuracies (UA/PA), F1 Score, and mean intersection over union (mIoU). We quantified predictive reliability via an entropy-based uncertainty measure (Section 2.3.5). For seasonal area statistics, we converted the predicted crop type outputs to hectares by counting pixels per class and multiplying by pixel area (10 m × 10 m = 100 m^2^ = 0.01 ha per pixel), reporting the total mapped area per season for each crop class.

#### 2.3.1 Satellite image processing in GEE

Both Sentinel-2 and Sentinel-1 data were processed in the GEE to build temporally aligned, biweekly stacks of the Wadi Al-Dawasir region and spanning the agricultural growing seasons starting in November 2022 and ending in October 2024. The Sentinel-1 and−2 datasets used in the GEE were from the analysis ready collections (S2 Level-2A surface reflectance; S1 GRD).

Sentinel-2 Level-2A products (S2_SR_HARMONIZED) provide surface reflectances after atmospheric correction (Sen2Cor), radiometric calibration, and terrain correction. We masked clouds and shadows using the Scene Classification Layer by excluding classes 0 (no data), 1 (saturated/defective), 2 (dark area), 3 (cloud shadow), 7 (unclassified), 8–9 (clouds), 10 (cirrus), and 11 (snow/ice) together with the cloud probability band set to less than 20%. Sentinel-2 images were aggregated into biweekly stacks consisting of 15-day timesteps using median values to stabilize the phenological signal. Doing this reduces the impact of residual clouds, haze and illumination changes, thus ignoring outliers such that the NDVI time series reflects the underlying crop signal rather than occasional contaminated pixels. The same procedure was applied consistently across years before computing NDVI for each timestep as (B8–B4)/(B8+B4) (Drusch et al., 2012).

To integrate SAR data, we used Sentinel-1 Ground Range Detected (GRD) scenes acquired in Interferometric Wide (IW) swath mode from the Sentinel-1 data collection (COPERNICUS/S1_GRD) filtered to the study area and the time periods of the field campaigns. We used dual polarization (VV, VH), allowing both ascending and descending passes. Processing included orbit correction and border/thermal-noise removal, radiometric calibration to the backscatter coefficient σ0 (the normalized radar cross-section of returned power per unit ground area), and terrain correction to reduce geometric distortions. We then converted the calibrated backscatter to decibels (dB) using 10 log10 (σ0). To reduce speckle while preserving field boundaries, we co-registered the processed Sentinel-1 imagery to the Sentinel-2 grid (i.e., same Coordinate Reference System, pixel size, and alignment). Temporal alignment was achieved by pairing each 15-day NDVI composite with the closest-in-time Sentinel-1 acquisition within ±6 days. When multiple acquisitions were available, we kept the nearest Sentinel-1 scene within ±6 days of the composite center date; if no acquisition fell within this ±6 day window, the radar value for that timestep was set to missing. We retained the VH/VV ratio in dB, rather than a single polarization, because this ratio emphasizes vegetation structure and volume scattering while reducing the influence of soil moisture and incidence angle variations, which is particularly important in irrigated arid environments such as Saudi Arabia (European Space Agency, n.d.; Veloso et al., 2017). Importantly, we used the VH/VV ratio, a commonly reported dual-polarization feature in Sentinel-1 crop classification studies, and retained this formulation for consistency of interpretation. Prior work shows that polarization ratios can improve crop discrimination relative to using VH or VV individually (Tsuchiya and Sonobe, 2025).

#### 2.3.2 Pseudo labels

To augment the field verified crop type labels, we produced additional pseudo labels using unsupervised K-means clustering, which is predefined in GEE. K-means clustering was selected because it is a simple and common unsupervised approach that does not require labels during clustering, which makes it suitable when field annotations are limited (Wang et al., 2019). We used full seasonal time series stacks for each season (biweekly Sentinel-2 NDVI paired with Sentinel-1 VH/VV) for every pixel. Clustering was restricted to an agricultural mask of seasonal median NDVI values > 0.5, in order to exclude natural vegetation, desert, and other built-up areas. We selected the number of clusters to be 8 (K = 8) after scanning K = 4 to 16. Candidate K values were evaluated using agreement with the available training field labels, based on overall accuracy and macro-F1, and cluster purity, based on Shannon entropy of the field-label distribution within each cluster. Lower entropy indicated clusters dominated by one crop group, while higher entropy indicated mixed clusters. K = 8 provided the best balance between field-label agreement and cluster purity and was therefore retained for pseudo-label generation in this dataset. These metrics were used only to guide the choice of K and were not intended to represent a universal setting. The selected K value is dataset-specific and depends on crop composition, field structure, and phenological variability. In other regions, K should be re-evaluated using limited local reference data, expert interpretation of temporal cluster profiles, or unsupervised cluster-validity diagnostics.

Clusters were then assigned to crop groups by identifying, for each cluster, the crop label most frequently observed within that cluster's pixels inside the verified field polygons. To reduce noisy pixels and encourage within-field consistency, i.e., more uniform labels within each center pivot field rather than scattered pixel-wise fragments, we retained only connected regions ≥ 100 pixels, about 0.1 ha, as a conservative minimum mapping unit at 10 m resolution to remove small fragmented patches that are more likely noise than true field-scale crop. Areas outside the NDVI-based agricultural mask, and noisy patches within fields, were treated as ambiguous and left unlabeled. The K-means derived pseudo labels were used to augment our field labels to provide additional supervision for the model during the fine-tuning stage, explained in section 2.3.3. The pseudo labels were not used for pre-training, validation, or selecting the model, which relied exclusively on the collected field labels. Since the pseudo labels were derived from unsupervised clustering rather than direct field verification, they were likely to contain some classification errors, particularly for crop types with overlapping temporal and spectral signatures or limited representation in the study area. The possible uncertainty and effect on fine-tuning should therefore be considered when interpreting the results. The K-means derived pseudo labels were used to augment the field labels and provide additional supervision during fine-tuning, as explained in Section 2.3.3. The pseudo labels were not used for pretraining, validation, testing, or model selection, which relied exclusively on the collected field labels. K-means clustering itself was applied to unlabeled seasonal Sentinel-2 NDVI and Sentinel-1 VH/VV time-series pixels. Limited field labels were used only to guide the choice of K and assign resulting clusters to crop groups within the training data. The pseudo labels were therefore used as an intermediate supervision source to increase label density during fine-tuning, not as a replacement for field labels or as part of the validation process.

#### 2.3.3 Pre-training phase

A masked autoencoder (MAE) is a self-supervised model that masks a portion of the input and trains an encoder and a decoder to reconstruct the masked content, resulting in transferable representations (He et al., 2022). In the pre-training stage, a MAE was trained to learn crop phenology characteristics directly from satellite image time series without labels. Each training sample was a season-specific sequence of biweekly Sentinel-2 derived NDVI paired with Sentinel-1 VH/VV, stored per modality as tensors of shape (T × H × W), where H=W=128 are the spatial dimensions in pixels, and T≈13, which represents the timesteps. NDVI values were clipped to a range of (−1, 1) and VH/VV to (−30, 0) dB to stabilize training. Because image acquisitions were irregular, the collate step selected a contiguous, aligned window of T = 12 shared by NDVI and VH/VV, where each frame corresponds to a 2-D median composite at a single 15-day timestep. Missing frames were zero-padded and masked so they did not contribute to the loss and were treated as no-data downstream.

**Figure 6:**
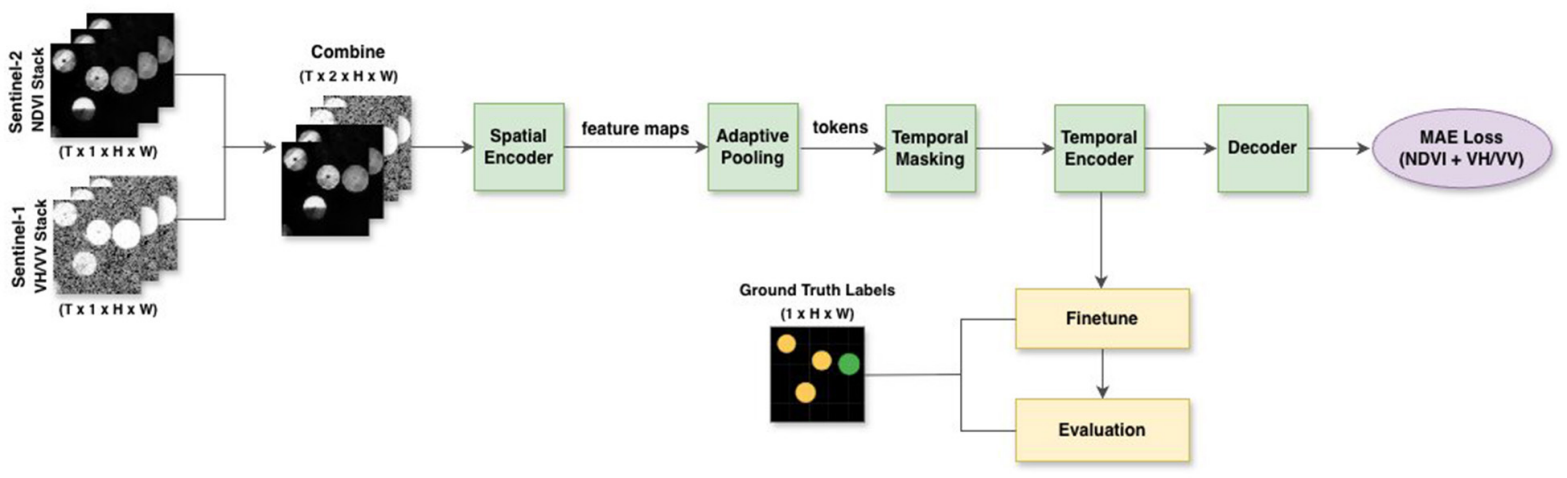
Pre-training pipeline (MAE-style) for NDVI and SAR streams. Each frame is encoded spatially, pooled to temporal tokens, randomly masked along time, encoded by a temporal transformer, and reconstructed by a modality-specific decoder. Reconstruction loss is computed only on masked timesteps.

The input NDVI and VH/VV data were combined as a two-channel input and processed jointly through a shared encoder, as illustrated in Figure 6. For each sample, the stacked inputs were passed through a small 3-layer spatial encoder (convolution, group normalization, and the Gaussian Error Linear Unit; GELU), which produced a feature map for each timestep. Instead of global spatial averaging, feature maps were reduced using adaptive average pooling to a coarse spatial grid, where each cell summarizes a local region of the field to preserve limited spatial structure while controlling model complexity. The resulting features were reshaped into a sequence of spatiotemporal tokens, where each token represents one grid cell at one timestep. Positional encodings were added to the tokens, and random temporal masking was then applied, with 50% of frames masked during training. The masked token sequence was passed to a transformer-based temporal encoder (Bertasius et al., 2021), which modeled temporal dependencies across the season. Spatial context was retained directly within the tokenized representation through the coarse spatial grid. The pre-training stage was designed to prioritize learning temporal crop dynamics rather than preserving full-resolution spatial texture. Spatial information was compressed rather than discarded, and the decoder incorporated a spatial prior derived from encoder feature maps during reconstruction. This design reduced model complexity while retaining sufficient within-tile structure to support temporal representation learning using limited labels and irregular phenology. Fine spatial detail and field boundaries were subsequently recovered during supervised fine-tuning with DeepLabV3, which explicitly exploits spatial context and refines segmentation boundaries.

During training, masked timesteps were removed from the input sequence, and the model learned to infer their representations from the remaining timesteps. The transformer produced context aware tokens at masked positions by aggregating information across the temporal sequence (self-attention). The reconstructed tokens were then passed to the decoder for reconstruction following MAE principles (He et al., 2022). To preserve spatial details, the decoder received a spatial prior defined as the mean of the encoder feature maps computed over the unmasked timesteps, resulting in a coarse spatial feature map. The spatial prior was fused with the token-based features and subsequently upsampled to 128 × 128 pixels for reconstruction.

We used the Huber loss (δ = 0.5), which behaves as squared error for small mistakes but transitions to absolute error for larger ones. This makes training stable while being robust to outliers or noisy data. The parameter δ sets the switch point, meaning errors smaller than 0.5 are penalized quadratically to encourage precise fits, and errors larger than 0.5 are penalized linearly to limit the impact of bad frames. We computed a Huber loss on the masked frames for each input channel, L_NDVI_ and L_VH/VV_, and then we optimized their sum as L_total_ = L_NDVI_ + L_VH/VV_. Summing the two losses trains the encoder on both signals at once, so the learned representation can capture how optical phenology and radar backscatter co-vary over time. This setup converged stably in practice. Training was conducted for approximately 500 epochs using a dataset split into training and validation sets (80/20), which gave us 11,721 training samples and 2,931 validation samples. A batch size of 4 was used due to GPU memory constraints, and optimization was performed using the Adam optimizer with a learning rate of 1 × 10^−4^.

The decoder (reconstruction) stage forces the encoder to model crop growth dynamics by predicting masked observations from temporal context. After pre-training, the decoder was discarded, and the encoder was then fine-tuned with a small labeled set for downstream crop mapping, following the standard practice in self-supervised learning approaches applied to image data (Ayush et al., 2021; Rußwurm and Körner, 2020).

#### 2.3.4 Fine-tuning phase

**Figure 7:**
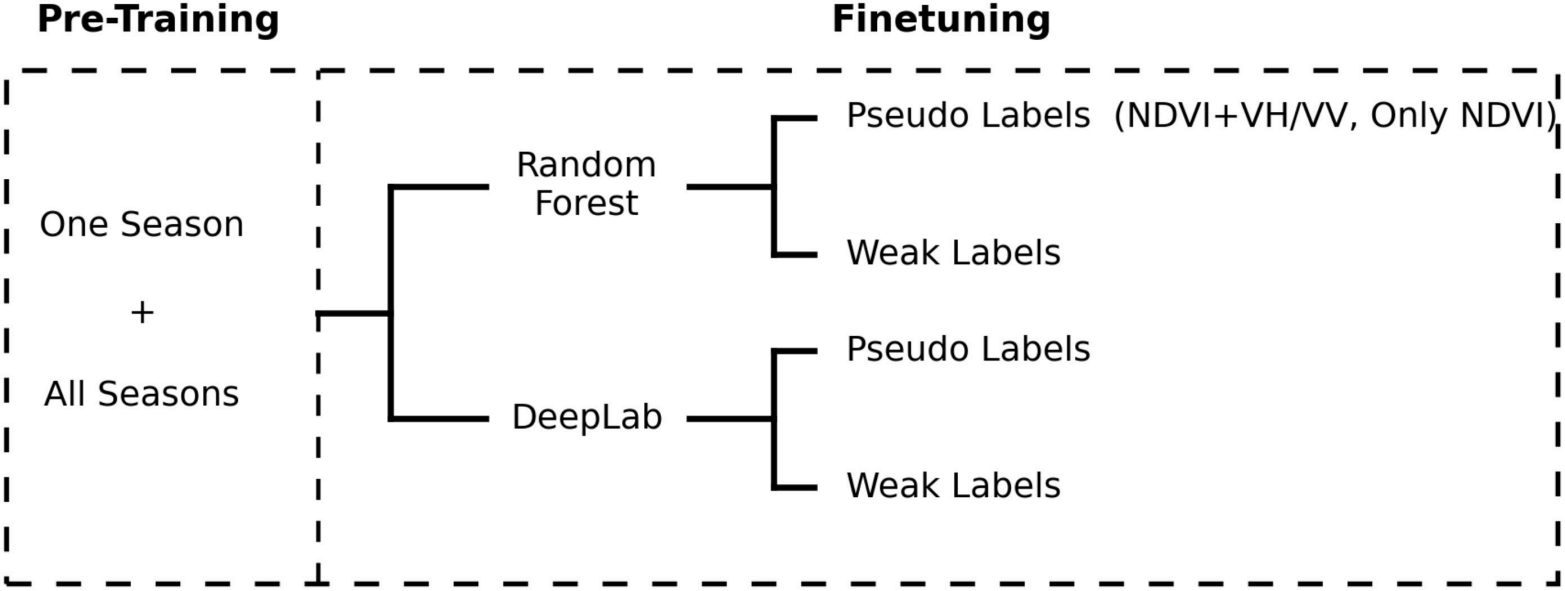
Experimental configurations used in the study. After masked-autoencoder pre-training, we compare two pre-training scopes (single season versus multiple seasons) and two supervision regimes for fine-tuning (verified field labels versus K-means-derived pseudo labels). Each configuration is evaluated using two downstream models: a DeepLabV3 segmentation model and an RF classifier.

Different experimental configurations were explored for fine-tuning the pre-trained encoders in order to evaluate the effects of pre-training scope, model of choice, and input modality (Figure 7). Encoders were initialized from the MAE pre-training stage. The two trained encoders with different scope configurations were either using data from a single season or all seasons.

For the supervised stage, we trained and evaluated two independent downstream models: DeepLabV3 and RF. DeepLabV3 was used as a semantic segmentation model with class-weighted cross-entropy loss, early stopping on validation loss, and gradient-norm clipping (1.0). It was selected because the target output is a spatially coherent field-level crop map, rather than only independent pixel-wise labels. The segmentation setup allows the model to use spatial context during prediction and to better preserve center pivot field structure (Chen et al., 2018). A RF model was trained at the pixel level on frozen MAE encoder features. For the RF approach, the NDVI and VH/VV stacks were passed through the pre-trained MAE encoder to obtain per-pixel embeddings at the native resolution, and the RF was trained on these embeddings using 600 trees, class-balanced training, and out-of-bag probability estimates. RF was included as a widely used remote-sensing baseline and as a comparison to evaluate whether frozen MAE features could support crop mapping without semantic segmentation (Belgiu and Dragut, 2016; Breiman, 2001).

Each configuration was evaluated with NDVI and VH/VV stacks as inputs. Only the NDVI stack was used as input for ablation. To test the effect of the amount of label density (supervision), we toggled pseudo labels (Sec. 2.3.2) on and off for the best configuration of downstream tasks and modalities. As noted earlier, pseudo labels were used only during training, and never for validation or testing. Moreover, we denoted configurations as Sn-r; where S represents season, n is the number of seasons used in pre-training, and r is the supervision type used for fine-tuning i.e., S1-F = single season pre-training + Field labels; S1-P = single season + Pseudo labels; S4-F = all seasons pre-training + field labels; S4-P = all seasons + pseudo labels.

#### 2.3.5 Evaluation data and metrics

We withheld 20% of labeled fields as a validation set, stratified by season and class at the field level, to provide an independent evaluation with sufficient representation across crop groups while retaining most labeled data for model development. Within the remaining 80%, training and validation were split 80/20 by field IDs for early stopping and model selection. The withheld validation set was never used for hyperparameter tuning or pseudo label mapping. Pseudo labels were generated only based on the training partition.

For accuracy assessment, we calculated the mean Intersection over Union (mIoU) and per class IoU, overall accuracy (OA), producer's accuracy (PA/recall), user's accuracy (UA/precision), and F1 per class. We also produced a confusion matrix. Metrics were computed separately for winter and summer and then summarized across seasons by averaging. In addition, to contextualize the performance of the proposed framework, we evaluated two external MAE-based foundation models, Prithvi 1.0 and ScaleMAE, under the same study protocol. For these benchmark experiments, we used the same label partitions, single-season inputs, supervision settings, and evaluation metrics, and tested both decoder-only adaptation and full fine-tuning. These comparisons were intended to provide a controlled reference for relative performance across architectures and training configurations under the same study protocol, rather than an exhaustive model-specific optimization. Therefore, the benchmark results should be interpreted as performance under the tested adaptation settings, not as the maximum achievable performance of Prithvi-EO-1.0 or ScaleMAE.

Uncertainty was quantified using normalized predictive entropy. Entropy summarizes the full probability vector to range from 0 to 1, where values near 0 indicate a confident decision and values near 1 indicate uncertainty. Scene level maps of confidence and entropy were displayed, and entropy was also overlain on an NDVI background to localize and interpret uncertainty to evaluate whether high entropy coincided with low vegetation signal, parcel boundaries, subpixel heterogeneity, or irrigation driven phenological transitions. These maps helped diagnose classification errors, guide targeted reference data collection, and highlight conditions that reduce performance.

For practical use, a low-confidence flag was defined by thresholding the DeepLabV3 confidence map (e.g., ≤ 0.50). Flagged pixels typically occurred along field edges, within mixed or partially harvested pivots, or on dates with very low vegetation activity in terms of NDVI signal. Conversely, very high confidence (e.g., ≥ 0.95) identified stable field cores that were suitable for area aggregation. While these confidence results were used to interpret the model predictions and guide quality control and future labeling, they were not used to modify labels in this study.

To extend uncertainty analysis to aggregate statistics, crop area totals were also estimated using class probabilities rather than only hard labels. For each class, expected area was computed as the sum of per-pixel class probabilities multiplied by pixel area. An approximate uncertainty term was derived using the Bernoulli variance, *p*(1−*p*), where *p* is the predicted probability for a crop class. This quantifies pixel-level uncertainty, which was then aggregated and summarized as standard deviation and confidence intervals for each crop area. This provides a simple estimate of uncertainty in crop area totals based on pixel-level probabilities, but it does not capture spatial correlation or systematic model errors.

## 3 Results

### 3.1 Temporal crop responses from Sentinel-2 NDVI

**Figure 8:**
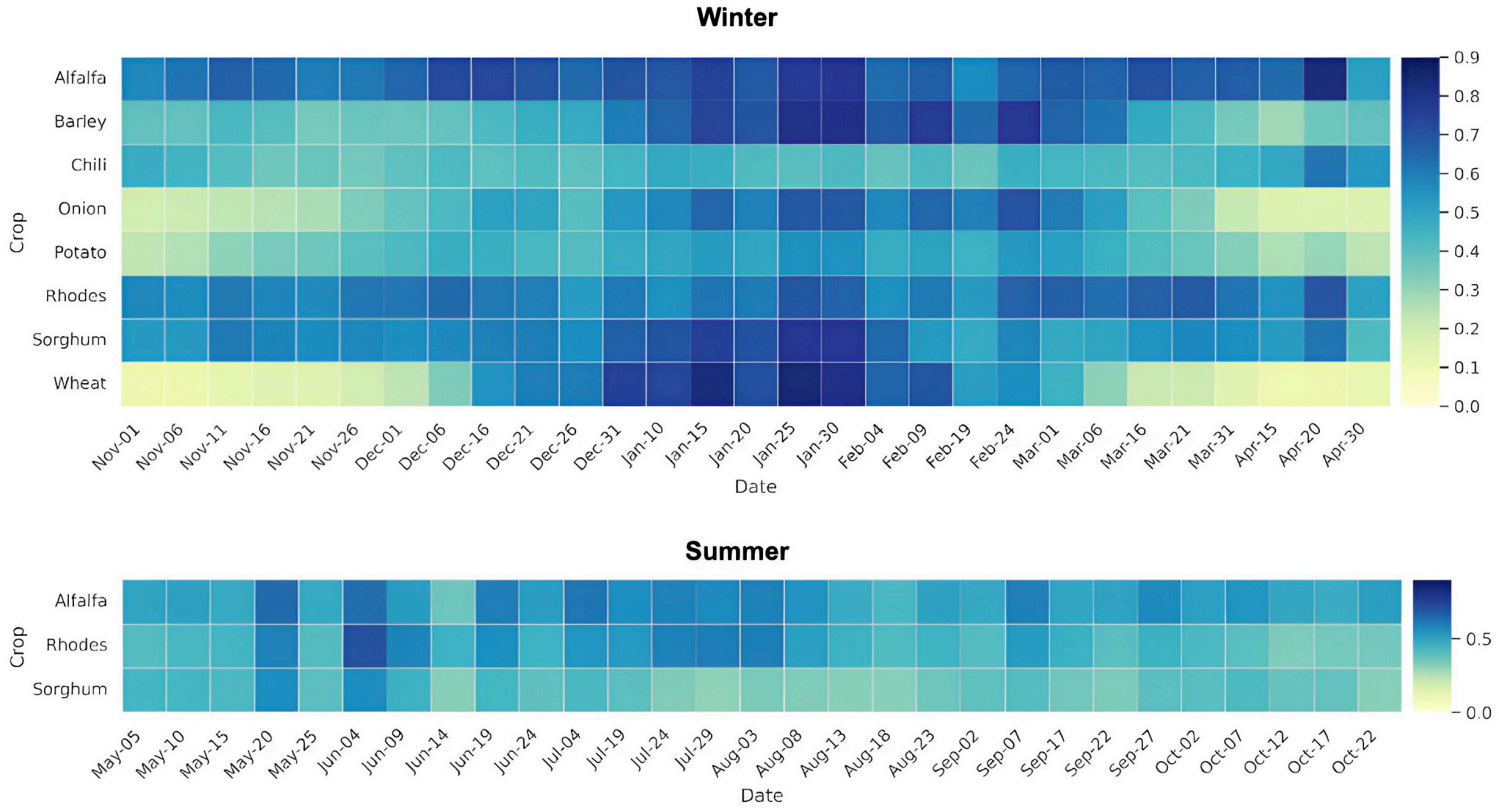
Heatmaps of mean temporal NDVI profiles of major crops during winter and summer seasons across all campaigns.

Temporal NDVI profiles derived from Sentinel-2 images revealed phenological patterns that help differentiate crop types. Figure 8 summarizes the mean seasonal NDVI trajectories per crop as heatmaps across labeled fields. Cereals and vegetables (e.g., wheat, barley, and potato) typically exhibited a single growing season with a rapid NDVI rise, a visible mid-season peak, and a decline, consistent with synchronized planting and harvest cycles. Forage crops (alfalfa and Rhodes grass) maintained elevated NDVI values for prolonged periods of activity throughout the year with multiple cuts and regrowth cycles, whereas other crops (e.g., sorghum, chili, and onion) showed more moderate and variable trajectories. Guided by these temporal behaviors, four functional groups (forage, cereal, vegetable, and fallow) were used for classification to maximize phenological separability at the field scale while maintaining sufficient samples per class for robust training and evaluation.

**Figure 9:**
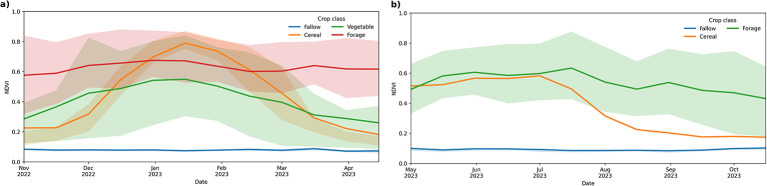
Seasonal NDVI trajectories by functional crop group showing mean trends (solid lines) and field-level variability (shaded bands), **(a)** winter season, and **(b)** summer season. Variability reflects differences in timing and magnitude of NDVI across fields, while functional groups retain distinct temporal patterns.

Because irrigation was not based on rainfall but rather on groundwater extraction, sowing and harvest dates can shift across fields and crop types. Figure 8 highlights the typical temporal patterns by crop based on mean seasonal NDVI profiles, whereas Figure 9 shows field-level variability within individual crop classes, particularly in the timing and magnitude of seasonal NDVI peaks. Nevertheless, the broader functional groups retained more consistent seasonal behavior than the individual crop classes. Therefore, to handle this variability, the full time series signal was used instead of fixed calendar thresholds, allowing the model to learn crop specific temporal trends from the NDVI and VH/VV time series. This grouping strategy was used to reduce label noise and improved class separability by collapsing similar crops into a smaller set of functional crop groups that remain more distinct at the field scale.

### 3.2 Overall performance across configurations: pre-training scope, pseudo labels, and model type

We evaluated the seasonal crop type mapping models across configurations that varied between the pre-training scope (single season vs. all seasons), pseudo label use (with vs. without), and model type (DeepLabV3 vs. RF), using NDVI along with VH/VV as the default input and NDVI only as an ablation, i.e., retraining the same architecture with identical settings but removing VH/VV, to isolate the contribution of SAR. We used an independent validation set that had neither been seen by the model during the pre-training nor fine-tuning stages for evaluation.

We observed that when using pseudo labels, the mIoU values increased from 43–45 when using field labels to 77–80 mIoU, using both the DeepLabV3 and RF models, with OA increasing from 91–92% to 96–97% and macro-F1 from 55–56 to 86–88. Pseudo labels increase the number of labeled pixels across fields and timesteps per season (Section 2.3.2), broadening spatial coverage and reducing class imbalance. This improvement was not uniform across crop groups. For fallow fields, performance was already strong under few-label supervision and pseudo labels provided a smaller additional gain, mainly reflected in spatial consistency. For cereals, pseudo labels improved precision by reducing overprediction of the cereal class, while recall changed more modestly. The largest gains were observed for vegetables and forage classes. Vegetables were previously poorly detected, while forage was frequently confused with cereals. With pseudo labels, both recall and mIoU increased substantially for these classes. Despite these gains, vegetables remained the most challenging class, with higher within-class variability and temporal overlap with cereals.

The DeepLabV3 approach slightly outperformed RF, specifically under the S4-P configuration, with mIoU of 80.1 for DeepLabV3 vs. 77.2 for RF (see Table 1). This difference is most apparent along pivot edges and during transition periods such as forage after cutting, newly sown or recently harvested cereals, and partially irrigated or abandoned pivots. With field-collected labels only (S4-F), the performance difference remained similarly small, with 45.0 vs. 42.0 mIoU for DeepLabV3 and RF, respectively.

**Table 1:** Results for four configurations that vary pre-training scope (1 season vs. 4 seasons), label type (field labels vs. field + pseudo labels), and model (DeepLabV3 vs. Random Forest).

Experiments				DeepLabV3					Random forest				
ID	Pre-training	Input data	Label type	OA	F1	PA	UA	mIoU	OA	F1	PA	UA	mIoU
S1 - F	1 season	1 season	Field labels	91.0	55.6	49.1	63.9	43.0	91.6	48.9	48.8	63.4	41.9
S1 - P	1 season	1 season	Pseudo labels	96.8	88.4	88.5	88.3	80.1	96.7	88.3	90.4	86.4	80.1
S4 - F	4 seasons	1 season	Field labels	91.7	56.0	51.0	64.0	45.0	91.0	50.0	48.6	61.2	42.0
S4 - P	4 seasons	1 season	Pseudo labels	96.9	88.3	88.2	88.4	80.1	96.4	86.1	87.8	84.6	77.2

Inputs were NDVI+SAR (VH/VV). Metrics are computed on the independent validation set.

The uncertainty analysis also establishes that DeepLabV3′s predictions are generally confident i.e. mean confidence 0.85 (median 0.88), with 16% of pixels at very high confidence (≥ 0.95) and only 5.2% at low confidence ( ≤ 0.50). The mean entropy is 0.29 (0 = confident, 1 = uncertain), which indicates low overall uncertainty. Most of the uncertainty occurs in the following cases. Higher entropy occurs over vegetable fields (potato, onion, chili) where crop timing overlaps with cereals, and over forage fields immediately after cutting and during short regrowth windows. Uncertainty is also higher for newly sown or recently harvested cereals, within summer fallow and partially irrigated or abandoned pivots, and along field edges where mixed pixels are common.

We also evaluated the selected S4-P DeepLabV3 setup (S4-P; DeepLabV3) using NDVI alone, i.e., excluding the VH/VV SAR feature. In this case, performance in OA dropped to 0.63 compared to 0.96 when using NDVI + VH/VV, and mIoU = 0.35 (from 0.80), UA = 0.39 (from 0.88), PA= 0.33 (from 0.88), and F1(UA,PA) = 0.31 (from 0.88). This sharp decline shows that excluding VH/VV reduced performance substantially. Therefore, NDVI combined with VH/VV remained the default input. Across configurations, pseudo label augmentation produced the largest performance gain, with mIoU increasing by roughly 35 points relative to training with field-labels only. Expanding the pre-training scope from one season to all seasons produced a smaller additional gain. These results indicate that, in this label-scarce setting, performance benefited most from adding intermediate supervision through pseudo labels together with the temporal optical–radar representation. The pseudo labels were used only during fine-tuning and were not used for pre-training, validation, or model selection.

We further compared the proposed framework against two external MAE-based baselines, Prithvi-EO-1.0-100M and ScaleMAE, under the same downstream crop-mapping task. Across all benchmark settings, neither Prithvi-EO-1.0-100M nor ScaleMAE achived the results of the proposed framework, as detailed in Table 2. The strongest comparison baseline was obtained with ScaleMAE using decoder-only training and field labels, which reached an mIoU of 46.8 and F1 of 63.48, followed by Prithvi also with decoder-only training and field labels, yielded an mIoU and F1 of 42.52 and 59.28, respectively. In contrast to the proposed framework, pseudo label supervision reduced performance for both external foundation models (used as baselines) across all tested settings, with mIoU decreasing to approximately 29–31. These results show that pseudo labels did not provide consistent benefit across model architectures in this study, which is further discussed in Section 4.

**Table 2:** Performance of MAE-based baselines under crop type mapping task.

Framework	Decoder	Fine-tuning	Label type	OA	F1	mIoU
Prithvi-EO-1.0	SegUperNet	Decoder-only	Field labels	89.30	59.28	42.52
Prithvi-EO-1.0	SegUperNet	Decoder-only	Pseudo labels	85.72	42.34	31.04
Prithvi-EO-1.0	SegUperNet	Full fine-tuning	Field labels	88.14	56.51	39.75
Prithvi-EO-1.0	SegUperNet	Full fine-tuning	Pseudo labels	85.91	42.42	31.13
ScaleMAE	SegUperNet	Decoder-only	Field labels	88.26	63.48	46.80
ScaleMAE	SegUperNet	Decoder-only	Pseudo labels	85.11	40.73	29.28
ScaleMAE	SegUperNet	Full fine-tuning	Field labels	87.36	55.97	38.99
ScaleMAE	SegUperNet	Full fine-tuning	Pseudo labels	84.39	41.13	29.44

### 3.3 Per class performance and errors

**Figure 10:**
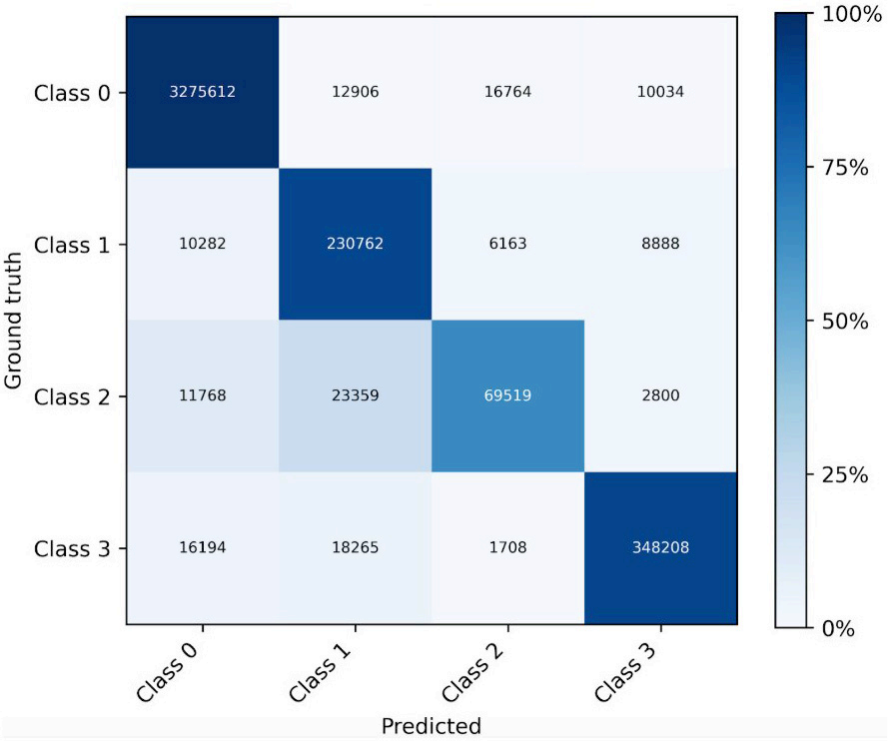
Confusion matrix for the best-performing model (DeepLabV3, S4-P). Class labels are fallow = 0, cereal = 1, vegetables = 2, and forage = 3.

Reporting on class-wise accuracies under the best configuration (S4-P; DeepLabV3; NDVI + VH/VV input) on the independent validation set, Table 3 shows that fallow and forage classes produce the highest mapping accuracies. The very high scores for fallow reflect a distinct signature of persistently low NDVI with limited variance, which is rarely confused with crop classes. Forage also shows high accuracy, with extended periods of elevated NDVI across the season. Cereal showed intermediate accuracy, with UA = 0.79, PA = 0.88, F1 = 0.85, and mIoU = 0.71 (Table 3). The cereal class exhibits a single seasonal peak that overlaps in timing and duration with vegetables. The higher PA than UA for cereal indicates that most true cereal fields are correctly identified, while some non-cereal fields are also predicted as cereal, resulting in lower precision. Vegetables were the most challenging crop group, as shown in Table 3. Figure 10 shows that the main error is vegetables being misclassified as cereals.

**Table 3:** Per class performance for the best performing model (DeepLabV3, S4-P, NDVI + SAR).

Per class	OA	UA	PA	F1	mIoU
Cereal	89.1	79.4	88.7	85.0	71.0
Fallow	98.0.	98.0	98.0	98.0	95.0
Forage	90.6	94.0	90.6	92.0	81.5
Vegetables	61.7	73.0	63.6	66.9	50.6

Metrics: OA, UA = precision, PA = recall, F1, mIoU.

The confusion matrix in Figure 10 summarizes misclassifications on the validation set for the best saved DeepLabV3 segmentation model. Rows denote ground truth classes and columns denote predicted classes, and values are row-normalized so each row sums to 100%. The diagonal represents per-class recall, and off-diagonal values show where pixels from a true class are assigned to other classes. Recall is highest for fallow and forage, indicating few missed pixels for these classes. Cereal also shows high recall, but some non-cereal pixels are predicted as cereal, contributing to commission errors and lower precision. The main confusion is vegetables being predicted as cereal, with a smaller fraction predicted as fallow, which corresponds to the lower F1 and mIoU for vegetables relative to the other classes.

**Figure 11:**
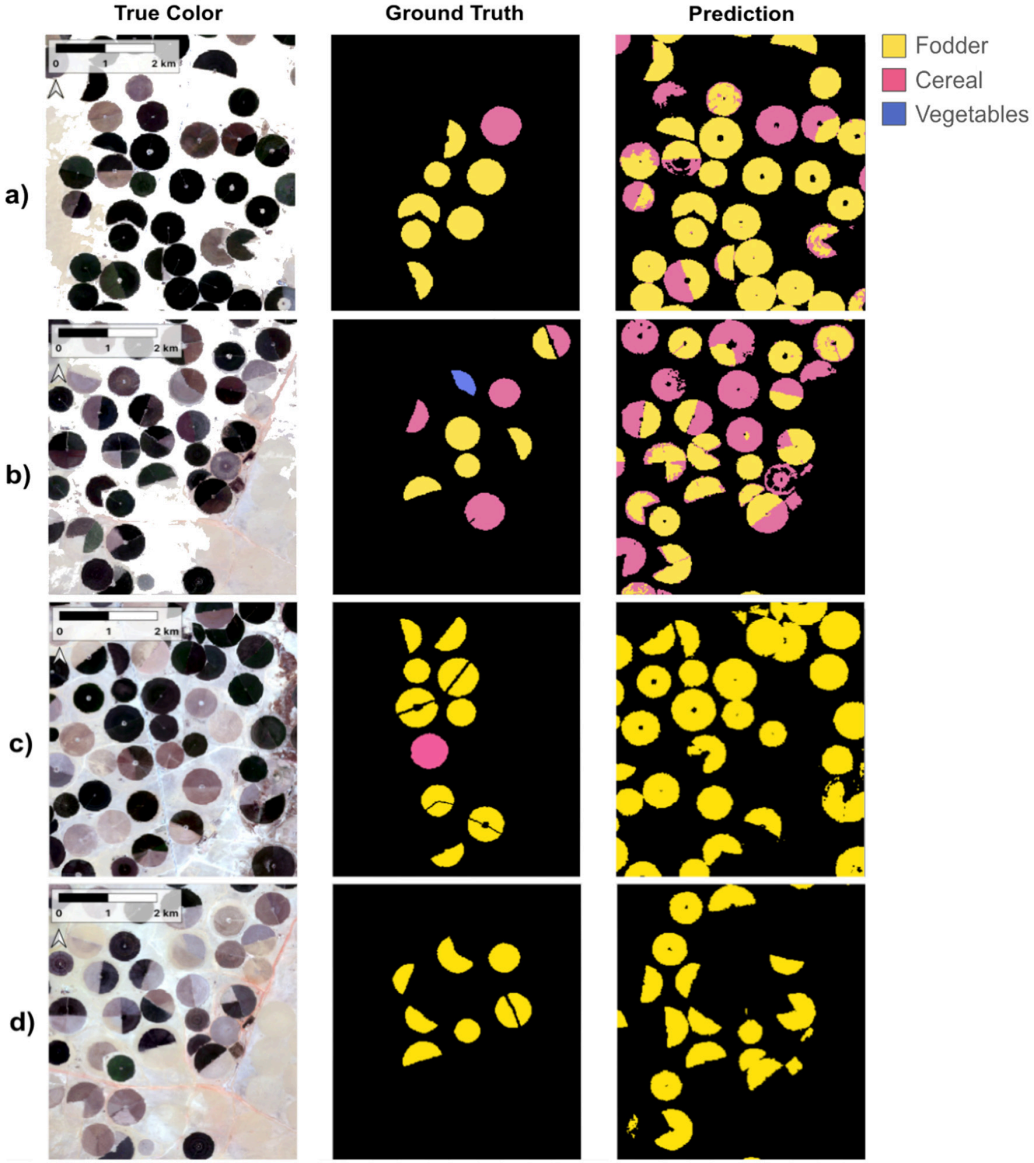
DeepLabV3 model performance across representative subsets. The RGB images **(left)** show agricultural center pivot patterns. Ground-truth annotations **(center)** include cereal (pink), forage/fodder (yellow), and vegetables (purple). Black pixels indicate fallow or background/unlabeled areas. The prediction map **(right)** shows the segmentation results from the self-supervised model at field scale.

The DeepLabV3 model was fine-tuned with data from the winter season of 2023 and then applied to the winter of 2024, summer of 2023, and summer of 2024. The resulting maps produced field-scale predictions that preserved center pivot geometry, with boundaries that follow field edges rather than fragmented pixel-wise patterns. In the winter maps, all four classes are present, and the main residual confusion occurs between cereals and vegetables, consistent with the per-class metrics. In summer maps, the label space is dominated by forage and fallow, and the maps reflect this with a clear separation of these two classes. Figure 11 shows that the segmentation outputs preserve center pivot geometry and field boundaries across scenes. In panels a and b, crop patterns are spatially consistent at the field scale, with most pivots assigned a dominant class rather than scattered pixel-wise labels. Residual errors are concentrated along field edges and partial pivots. The most noticeable confusion occurs between cereals and vegetables, while forage remains visually distinct and is mapped consistently across fields.

### 3.4 Crop area dynamics over time

We produced seasonal crop area estimates from the model outputs. After inference, predictions were masked to the agricultural footprint, a small inward buffer was applied at pivot edges to reduce mixed pixels, and pixels were aggregated per class and converted to hectares to report seasonal crop area totals by year. The time series of cultivated areas (Table 4) shows temporal changes in crop area distribution in Wadi Al-Dawasir between 2020 and 2024. Forage crops reached a peak area of 17,441 hectares in 2022 during the summer season, before declining to 8,775 hectares by summer 2024.

**Table 4:** Crop area estimates in Wadi Al-Dawasir (2020–2024) derived from the DeepLabV3+ crop maps.

Year	Winter forage	Winter cereal	Winter vegetables	Summer forage
2020	11,200	6,311	4,327	13,143
2021	13,000	8,277	2,534	14,315
2022	16,500	9,806	3,104	17,441
2023	16,600	9,766	3,053	13,361
2024	10,800	15,076	3,544	8,775

Cereal, forage, and vegetable areas are seasonal totals in unit hectare (ha).

Table 4 shows the progression of cereal, forage, and vegetable crop areas between 2020 and 2024. Cereal crops show an overall increasing trend, peaking in 2024. In contrast, forage crops increased until 2022 and then declined in subsequent years. Vegetable crop areas remained relatively stable over time. Seasonal patterns show that forage crops dominated summer agriculture, reaching peak extent in 2022, followed by a decrease in later seasons. Conversely, cereal crops expanded during winter seasons, reaching a maximum area of 15,076 hectares in 2024.

To complement the crop area estimates, uncertainty was propagated into crop area totals using class probabilities. The expected area for each class was computed as the sum of per-pixel class probabilities multiplied by the pixel area, rather than relying solely on hard class assignments. An approximate uncertainty term was derived from aggregated Bernoulli variance and summarized as standard deviation and confidence intervals. The resulting uncertainty bounds were small relative to the reported hectare values. For example, for winter forage, which ranged from 10,800 to 16,600 hectares across the study period, the associated uncertainty was on the order of ±10–12 hectares (< 0.1%). Similar magnitudes were observed for other classes, including winter cereal (6,311–15,076 ha) and summer forage (8,775–17,441 ha), where uncertainty remained below ±0.1% of the total area. These narrow confidence intervals indicate stable probability-based area estimates relative to the magnitude of total mapped area. However, these uncertainty bounds should be interpreted as approximate rather than comprehensive map-level uncertainty. The estimate is based on pixel-level class probabilities and does not explicitly model spatial dependence among neighboring pixels or fields. Agricultural fields are spatially structured, so prediction errors can be correlated within fields or repeated along field boundaries. Therefore, these uncertainty estimates provide useful context for interpreting temporal changes in crop area, but they do not represent a full spatial error propagation.

## 4 Discussion

The results show that limited label availability remains the main challenge in this crop type mapping task. The large improvement associated with pseudo labels indicates that expanding supervision across space and time was more effective than increasing pre-training scope alone in the proposed framework. Similar patterns have been reported in recent self-supervised remote sensing studies, where downstream performance depends more strongly on fine-tuning data than on pre-training scale (Ayush et al., 2021; Cong et al., 2022). However, the benchmark results show that pseudo labels did not provide the same benefit across all model architectures. In this study, the strongest results came from combining temporal representation learning, optical-radar inputs, pseudo labels, and segmentation based fine-tuning to produce spatially coherent field-level crop type maps with limited label availability.

The phenological behavior of crop types reveals a key limitation in irrigation driven agriculture. Fallow and forage were consistently separable because they showed distinct temporal signatures, while cereals and vegetables showed overlapping seasonal NDVI profiles. As shown in Figures 8, 9, winter cereals and vegetables followed similar single-season NDVI trajectories, with comparable timing of greening, peak vegetation growth, and senescence phases. The vegetable class added another source of difficulty because it grouped several crops, including potato, onion, and chili, into one category. These crops can differ in canopy structure, vegetation density, and NDVI magnitude. Together with the overlap between cereals and vegetables, this variability within the vegetable class explains why vegetables remained the least reliable class and why confusion between vegetables and cereals persisted in the validation results.

The misclassification of vegetables as cereals represented a meaningful limitation for certain applications. With an mIoU of approximately 0.50, vegetable predictions were less reliable at the field scale, particularly during winter seasons when phenological overlap was strongest. For applications requiring crop-specific estimates, such as targeted yield assessment or crop type specific policy enforcement, this level of accuracy may introduce uncertainty. However, for broader monitoring objectives, including distinguishing forage from non-forage systems or tracking seasonal shifts in cropping patterns, the impact is more limited because these tasks depend on separability between major functional groups rather than fine-grained crop classes such as alfalfa, wheat, or potato.

The contribution of SAR data was found particularly important. The strong performance reduction observed when using NDVI-only input indicates that optical signals alone did not sufficiently capture crop differences in irrigated arid environments. SAR backscatter provided complementary sensitivity to vegetation structure and biomass, which improved discrimination where spectral signals were ambiguous (Sun et al., 2020; Veloso et al., 2017; Weilandt et al., 2023). This is consistent with prior studies showing that multi-modal fusion is especially beneficial under non-standard phenological conditions. Moreover, the external benchmark comparison clarifies the role of model-task alignment in this study. Although pseudo label augmentation was the major source of improvement within the proposed framework, it did not improve performance for all models. Under the same study setting, pseudo labels reduced crop mapping accuracy for both Prithvi-EO-1.0-100M and ScaleMAE. This suggests that pseudo labels were most useful when they were consistent with the features learned by the model and with the target crop mapping task. The proposed framework learned representations from local Sentinel-1/Sentinel-2 time series in a hyper-arid, center pivot agricultural system. In contrast, Prithvi-EO-1.0-100M and ScaleMAE were pretrained externally on broader remote-sensing datasets, with different input structures, data distributions, and pretraining objectives. They were not designed specifically for hyper-arid irrigated agriculture or for the same NDVI and VH/VV temporal input used in this study. Therefore, the observed gain should be interpreted as the result of combining local temporal representation learning, multimodal inputs, pseudo labels, and segmentation-based fine-tuning, rather than pseudo labeling alone. A fuller diagnosis of the external baselines would require model-specific optimization and additional checks of training behavior, including learning curves, validation performance across epochs, and sensitivity to pseudo-label noise, to distinguish underfitting, overfitting, and representation mismatch.

Observed crop area dynamics further demonstrated the value of satellite-based monitoring. The decline in forage area after 2022, together with the increase in winter cereal area, indicates a shift in crop composition over time. These results do not establish causality. However, they are consistent with broader evidence that Saudi Arabia's irrigated agriculture has changed over time, including reported shifts in national center pivot extent and agricultural activity (Li T. et al., 2023). Satellite-derived time series therefore provide an independent way to track local crop area trajectories in places such as Wadi Al-Dawasir and compare them with broader national trends.

From an application perspective, the maps were most reliable for monitoring dominant crop systems, particularly forage, rather than for crop-specific accounting across all classes. Reliable identification of forage supports tracking water-intensive agriculture, which is relevant for irrigation planning, seasonal reporting, and regional resource allocation. Field-scale consistency also enables aggregation of mapped areas to quantify broad changes in crop composition over time. However, reduced mapping accuracy of vegetables limits the reliability of crop-specific interpretation. The maps were therefore deemed suitable for tracking forage dynamics and broader shifts in crop composition, but less suitable for applications that depend on accurate estimates of vegetable types. Crop type maps can support monitoring of major cropping changes over time, but they should not be used on their own to quantify vegetable production trends or other vegetable-specific assessments without further improvement of the vegetable class. The multi-seasonal mapping results demonstrate temporal applicability within Wadi Al-Dawasir, where the framework was applied across multiple growing seasons. However, spatial transferability to other agricultural regions remains untested. Transfer to other arid systems may be affected by differences in crop composition, irrigation practices, soil background, field geometry, and crop calendars. These factors can change both the temporal and spectral signals learned by the model. For example, different crop mixtures may introduce classes that were not represented in the Wadi Al-Dawasir training data, while different irrigation practices can alter greening, harvest, and regrowth patterns. Soil brightness, salinity, and field geometry can also affect NDVI and SAR responses, especially in fragmented fields or areas without regular structure of center pivot fields. Achieving spatial transferability would therefore require testing the framework in additional arid regions, supported by limited local reference data, region-specific calibration, or fine-tuning to account for local crop types, management practices, and background conditions.

## 5 Conclusion

In this study, we proposed a framework for crop type mapping in a label scarce setting, covering a hyper-arid agricultural region using multi-temporal Sentinel-1 and Sentinel-2 data. The developed approach integrates self-supervised representation learning, pseudo label augmentation, and multi-modal inputs to address limited labeled data. Results showed that increasing label density through pseudo labels contributed to the largest performance improvement, enabling the best configuration to reach ~0.80 mIoU, 0.88 F1, and 0.97 OA for the four crop classes, while the benchmark comparison indicated that such gains were not universal across pretrained encoders. These findings indicate that availability of labeled data, rather than temporal pre-training depth, remains the primary constraint in crop type mapping. These results directly address the study objective of developing a scalable crop mapping approach under limited labeled data conditions.

Class-wise performance reflects differences in phenological separability. Fallow and forage achieved high mapping accuracies due to distinct temporal signatures, whereas cereals and vegetables showed similar seasonal behavior, which led to persistent confusion and lower performance for vegetables (mIoU ≈ 0.50). NDVI variability analysis confirmed substantial within-class variation and overlap between cereals and vegetables under irrigation-driven conditions, where planting and harvesting are not synchronized across fields. These results indicate that classification difficulty is driven by both data limitations and similarities in crop growth patterns. Important limitations remain, i.e. minority-class performance, particularly for vegetables, limits reliable field-level crop type discrimination, the analysis was limited to one hyper-arid, center pivot agricultural region, and the optical input was restricted to NDVI to reduce input dimensionality and computational cost, which limited the model's ability to use additional spectral information from red-edge and shortwave infrared (SWIR) bands.

Future work should focus on improving separation between phenologically similar classes, particularly cereals and vegetables. Full multispectral Sentinel-2 inputs, including red-edge and SWIR bands, should be evaluated to determine whether additional spectral information improves discrimination beyond NDVI-based trajectories (Forkuor et al., 2018; Griffiths et al., 2019; Immitzer et al., 2016). Higher temporal sampling density and higher spatial resolution imagery could further reduce class overlap and mixed-pixel effects (Sun et al., 2019). Future work should also improve how classification uncertainty is carried into field-level crop type maps and area estimates, and evaluate spatial transferability across arid agricultural systems with different irrigation methods, soil backgrounds, field geometries, crop calendars, crop composition, and management practices.

Overall, the framework demonstrates practical potential for large-scale agricultural monitoring in water scarce environments. Reliable identification of forage systems in the case of Saudi Arabia enables monitoring of water-intensive cropping patterns, while seasonal crop maps enable tracking of temporal changes in cultivated area. These capabilities support applications in irrigation planning, crop monitoring, and resource allocation. The combination of representation learning and multi-modal satellite data provides a scalable pathway for crop type mapping in data-scarce regions.

## Data Availability

The original contributions presented in the study are included in the article/supplementary material, further inquiries can be directed to the corresponding author.
